# New Trends of Digital Data Storage in DNA

**DOI:** 10.1155/2016/8072463

**Published:** 2016-09-05

**Authors:** Pavani Yashodha De Silva, Gamage Upeksha Ganegoda

**Affiliations:** Faculty of Information Technology, University of Moratuwa, Katubedda, Moratuwa, Sri Lanka

## Abstract

With the exponential growth in the capacity of information generated and the emerging need for data to be stored for prolonged period of time, there emerges a need for a storage medium with high capacity, high storage density, and possibility to withstand extreme environmental conditions. DNA emerges as the prospective medium for data storage with its striking features. Diverse encoding models for reading and writing data onto DNA, codes for encrypting data which addresses issues of error generation, and approaches for developing codons and storage styles have been developed over the recent past. DNA has been identified as a potential medium for secret writing, which achieves the way towards DNA cryptography and stenography. DNA utilized as an organic memory device along with big data storage and analytics in DNA has paved the way towards DNA computing for solving computational problems. This paper critically analyzes the various methods used for encoding and encrypting data onto DNA while identifying the advantages and capability of every scheme to overcome the drawbacks identified priorly. Cryptography and stenography techniques have been analyzed in a critical approach while identifying the limitations of each method. This paper also identifies the advantages and limitations of DNA as a memory device and memory applications.

## 1. Introduction

The excursion of data storage initiated from bones, rocks, and paper. Then this journey deviated to punched cards, magnetic tapes, gramophone records, floppies, and so forth. Afterwards with the development of the technology optical discs including CDs, DVDs, Blu-ray discs, and flash drives came into operation. All of these are subjected to decay. Being nonbiodegradable materials these pollute the environment and also release high amounts of heat energy while using energy for operation [1].

With the employment of digital systems for the purpose of generation, transmission, and storage of information, there rises a need for active and ongoing maintenance of digital media. With the massive amounts of digital data that has to be stored for future use, a problem arises in the storage of irresistible amounts of data. The demand for data storage is rapidly increasing day by day. The total information storage of the entire world was around 2.7 ZB in 2012. Every year the storage necessity is increasing by 50% [2]. Currently almost all of the digital data is stored with a technology that will last only for a limited time period. Memory cards and chips are sustainable for 5 years from their preliminary use [3]. Standard hard drives are prone to damage from high temperatures, moisture, and exposure to magnetic fields and through mechanical failures. Though solid state drives operate better than hard drives, if not power-driven for more than few months they tend to lose their information [3]. Therefore researchers' devotion has been driven towards development of a storage mechanism which overcomes the aforementioned drawbacks successfully.

Taking into account the manner fossil bones preserve genetic material for ages, researchers paid their attention towards using deoxyribonucleic acid (DNA) as a storage medium.

DNA has an unbelievable storage capacity. Castillo states that all the information in the entire Internet could be located in a device which is lesser than unit cubic inch [3]. DNA is witnessed as the optimal medium in this regard fundamentally because instead of using 1 s and 0 s by the computer to store data, DNA consisting of adenine, guanine, cytosine, and thymine (A, G, C, and T) already paired into nucleotide base pairs A-T and G-C can be utilized for storing information in a form of binary code [1]. As the urgent need for high capacity storage medium rises, DNA is considered ideal in this regard as single nucleotide can represent 2 bits of information. Accordingly 455 EB of data can be encoded in 1 gram of single stranded DNA (ssDNA) [3]. Entire information that is produced by the world over a year can be stored in just 4 grams of DNA [1]. High memory space is offered by DNA as it is 3-dimensional (3D) by structure. DNA offers readable and reliable information for millennia, which can be extended to almost infinity by drying and protecting from oxygen and water [3].

DNA can withstand a broader range of temperatures (−800°C–800°C). It utilizes power usage million times more effectively than a modern personal computer. Additionally it privileges more storage options as it stores data in a nonlinear structure unlike most of the media storing data in a linear structure. DNA promises more options to improve latency and extraction of data, as it allows reading data in bidirections. The important fact that DNA is invisible to human eye ensures that DNA is secure and is impossible to be harmed by living organisms [1].

This research paper critically analyzes the techniques used in writing and reading data into DNA. Many models of encoding were used to encode data into DNA. First section of this review paper provides a background for digital data storage in DNA, analyzing the perfection of DNA to be used as a storage medium. The second section of this review paper analyzes the results and methods of the models used for encoding data into DNA while critically analyzing the custom in which each model overcomes the drawbacks identified in the prior models. In view of the organization of the first subsection of the second section, function of DNA as a storage device is highlighted in this. Evolution and development of encoding models in the recent past have been summarized firstly in this subsection. Secondly encryption schemes used for encryption of data in DNA have been discussed in detail, diagnosing their significant features, advantages, and drawbacks associated. Thirdly, several approaches used for designing of codons have been discussed. Basic data storage styles have been analyzed in a critical manner by identifying the advantages over the other finally in the first subsection. The second subsection of Section 2 discusses the applicability of DNA storage for secret writing in the fields of cryptography and stenography. Considering the structure of the second subsection, firstly problems associated with DNA cryptography and stenography have been discussed in detail while this paper critically analyzes DNA secret writing algorithms by identifying the advantages offered from each technique and the drawbacks associated with each technique while suggesting the possible approaches to be implemented to overcome the associated drawbacks secondly. Approach of DNA as an organic memory device and possible applications of DNA as a memory device have been discussed in the third subsection of Section 2. The fourth subsection of Section 2 of this article describes in detail manner in which big data storage and big data analytics have paved the way towards DNA computing. Approaches undertaken to solve computational problems employing a DNA computer and possibility of DNA computer to massively use its vast parallelism effectively are discussed here along with the drawbacks associated with each approach.

## 2. Materials and Methods

### 2.1. DNA as a Storage Device

Two lengthy strands of nucleotides together compose a DNA molecule. Each nucleotide contains one of four bases (A: adenine, G: guanine, C: cytosine, and T: thymine), along with a deoxyribose sugar and a phosphate group. DNA molecule comprises a double stranded structure, comprising two single stranded sets of nucleotides bonded by A=T double hydrogen bond or C≡G triple hydrogen bond. These two single strands which are bonded together by hydrogen bonds are referred to as complimentary strands. A single stranded DNA is positioned in between two ends 5′ (5 prime) and 3′ (3 prime) [4]. Information is packed into chunks of nucleotides called “oligonucleotides” as accumulating of lengthy DNA strands is problematic [3]. Process of synthesizing using a DNA synthesizer obtains single stranded DNA chains, artificially composed of about 50–100 oligonucleotides. Hybridization is the process of individual single stranded DNA (ssDNA) forming double stranded DNA (dsDNA), coupling with complementary ssDNA under certain conditions. Ribonucleic acid (RNA) is a ssDNA comprising ribonucleotides in which thymine (T) is replaced with Uracil (U) [4].

**Figure 1:**
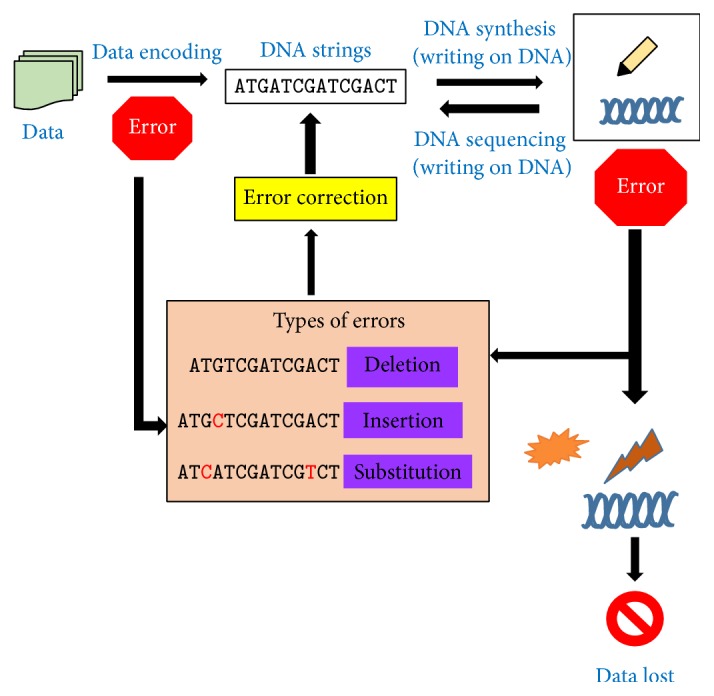
Schematic representation of storing data on DNA.

Information is usually read in base pairs as DNA is double stranded. Currently with the aid of new DNA synthesizing techniques the way in which base nucleotide pairs are generated is being reformed because it is a challenge to read G-C pair consecutively. Traditional base pairs were A-T and G-C. Innovative base pairs, A-C and G-T, are being used by newer technologies. A-C is employed to code 0 while G-T is used for 1 [4].  Figure 1 diagrammatically represents the general procedure of storing data on DNA.

#### 2.1.1. Encoding Data on DNA

*Microvenus Project.* This project was initiated by Joe Davis to store an image in DNA, which had the core intention of storing abiotic data in DNA. Encoding was based on the molecular size of bases, C → 1, T → 2, A → 3, and G → 4. Each and every nucleotide was assigned a phase structure C → X, T → XX, A → XXX, G → XXXX.

Encoding was achieved by placing a nucleotide at each repeated position of 1 and 0 bits, for instance, 100101=CTCCT and 10101=CCCCC. In the process of decoding, “C” could be decoded as “1” or a “0,” because only the number of repeated bits was taken into consideration at the time of encoding. For instance, CTCCT could be decoded as 011010 or 100101. Therefore this scheme of encoding was inaccurate as it was not distinctively decodable [5, 6].

*Genesis Project.* This was introduced by Eduardo Kac. He created an “artist's gene,” a synthetic gene by converting a sentence from the bibliographical book “Genesis” into Morse code and then converting the Morse code into DNA base pairs [7]. Hyphen and full stop were represented by bases T and C while replacing word space and letter space with A and G, respectively [5, 7]. Synthetic genes were fused into bacteria and in the presence of an ultraviolet light, mutations were caused in bacteria.

These mutations have in turn caused changes in the sentence. When the genes were decoded back into the Morse code and then back into English, the original sentence has been changed [7]. Genesis project was inaccurate as the original sentence was altered during mutation at the presence of ultraviolet light.

These 2 methods laid the foundation for encoding in DNA but were inaccurate as it was not uniquely decodable and the original content was altered due to mutations in the Microvenus and Genesis projects, respectively.

*Polymerase Chain Reaction Based Encoding Schemes.* In this method the data sequence is converted into a DNA sequence using a rule, which is an encryption key or a “codon.” The encoded DNA sequence is placed in between two unique template DNA regions corresponding to the forward and reverse primers. dsDNA is prepared according to the design of the DNA encoded message and is inserted into genomic DNA. In information readout process, amplification is done by Polymerase Chain Reaction (PCR) and is decoded by DNA sequences [8].

Limbachiya and Gupta used microdots to store data. This approach was secure because of its size and even if an adversary identifies the microdot, it would be extremely difficult to read the data without the knowledge of the primer sequence. But the limitation was scalability of data encoded in the limited size of microdots, as only 136 bits of data could be encoded [5].

To overcome this limitation Kac proposed information DNA (iDNA) [7] which consists of single Polyprimer Key (PPK), forward and reverse primer common 5-6 bases, to indicate the stored information. PPK functions as the data location identifier. In data encoding ternary code is being mapped to 3 bases (A, C, T) while sequencing primers consist of 4 bases with the 4th position as G in order to avoid mispriming [9]. In decoding process PPK is decoded first with the intention of decoding forward and reverse primers and then based on a unique sequencing primer can retrieve the information.

Main advantage of PCR based encoding models is high security as the recipient should be aware of the encryption key and primer. Hitches associated with PCR methods are the need of PCR, need of the knowledge of primers, widespread experimental obstacles, and practical problems. Insertion of errors in template region makes recovering the encoded data unmanageable. Moreover data breakage could occur in encoding and decoding procedures due to errors of humans [10].

As a result researchers paved their attention towards developing PCR independent data encoding models.

*Alignment Based Encoding Models.* This model was PCR independent. Alignment based approach introduced a data storage and retrieval method based on sequence alignment of DNA. Specialty of this approach is that this performs retrieval without parity checks, DNA template, or error correcting algorithms [11].

Yachie et al. proposed a method to copy and paste data within a sequence of an organism to achieve flexibility of storage and vigor of data inheritance, mostly appropriate in using DNA as trademarks/signatures of living modified organisms (LMOs) and as valuable transmissible media [12].

Through this approach it is possible to retrieve data from a living DNA without additional material such as template DNA, and it needs only the sequencing of the complete genome.

Data retrieval by PCR based amplification is prone to breakage of either side of DNA annealing sites, which are crucial for reading even parts of encoded data [13].

Advantages of this approach are greater speed and lower cost of reading DNA data and lower cost of synthetic DNA. This approach ensures higher durability and data inheritance as multiple copies of data are available and each copy is capable of detecting and correcting errors of the other copy [12].

Data could be lost during evolution. In order to prevent this Yachie et al. introduced different nucleotide sequences encoding the same data by multiple data compression paths [12].

Disadvantage of this approach is that multiplication of cassettes leads to redundant volumes. Parity effects cost a certain volume of data sequence; at the same time data recovery rate is fragile and is proportional to data breakage which occurs through DNA deletion of long ranges. Positions of the data breakages could be identified easily by the alignment results although they were not recoverable [12].

Main downside of this approach was the size limit of the cassette oligonucleotides being used to encode the message. If it increases a certain limit there is a possibility of it to appear by chance in host genome. And also sequencing of the entire genome is required to retrieve data. Thereafter Alienberg proposed improved Huffman coding method in which nucleotides were used efficiently and used specific primers for different types of files. Improved Huffman coding defines DNA codes for the entire keyboard, for clear-cut information coding. This is based on a construction of a plasmid library with specially designed primers embedded along with the message for fast retrieval. A good encoding scheme should have economical use of nucleotide per character which is about 3.5 here [12, 14].

*Rewritable and Random Access Based DNA Storage System.* Main features of this architecture are the random access to data blocks of DNA which promotes nonlinear access and rewriting capability of information into random locations. This approach successfully addresses the drawback of the existing approaches, the need to read the entire file of data to read only one fragment of data. And also the existing methods are read-only methods whereas this approach is rewritable. Undesirable cross hybridization problems are eliminated in this method by prohibiting redundancy of information.

Tabatabaei Yazdi et al. encoded Wikipedia pages of six universities, carefully chose parts of the stored data, and edited text written into DNA related to three universities. Shifting from current read-only methods to rewritable methods requires to address the below mentioned drawbacks [13].

- There is need to rewrite the entire content in order to edit in a compressive domain.
- Fourfold coverage is used to ensure reliability of information which makes the rewriting process much complicated because in order to rewrite one base modification of four locations is needed.
- Addressing method is utilized only to read the position of a read but does not perform selective reads.

This method can be effectively utilized for accessing random data sections and also for storing frequently updated data which needs to memorize the editing history [13].

Blaum et al. used DNA sequences consisting of special strings of addresses to access random information. These DNA sequences are also encoded with error correction mechanisms. Mutually noninterrelated addresses are designed while they satisfy error-control running digital sum constraint [15]. Encoding is done through threading together prefixes of addresses which are accurately concluded. As addresses are noninterrelated and chosen with a considerable hamming distance there is a very less probability for the addresses to be tangled with each other. Prefix encoding format [16] was chosen to select blocks to be rewritten while gBlock [17] and Overlap Extension PCR (OE-PCR) [18] methods were used to accomplish rewriting. Sanger sequencing [19] is used for decoding.

gBlock [17] method is more efficient but needs long and expensive primers for sequencing. OE-PCR method [18] uses cheap and short primers but is done in several steps.

High cost is one of the drawbacks of this approach. Instead of using Sanger sequencing employing next generation sequencing methods [19] will drastically reduce the cost of readouts. 

*Next Generation Digital Information Storage.* Microvenus project [5, 6], Genesis project [5, 7], PCR based encoding models [11], and alignment based encoding models [12] only stored small amounts of data. Hence this model proposed an efficient one-bit-per-base algorithm. In this approach Church et al. improved a scheme to encode arbitrary information employing next generation DNA synthesis and sequencing technologies. A draft of html coded book with 53,426 words, 11 JPG images, and 1 JavaScript program is being encoded. This encompasses a 5.27 MB stream [20].

Primary advantages of this approach over the prior approaches used for encoding include the employment of one-bit representation per base (G or T for 1 and A or C for zero). This brings in the capability of encoding sequences which are challenging to be read or written due to containing repeats, secondary structures, and extreme GC content. As we are splitting the stream of bits into data blocks thereby Church et al. are avoiding constructing of long DNA which is challenging to assemble in reading the information. Synthesizing, storing, and sequencing multiple copies of olingo are done with the intention of evading sequence verifying constructs and cloning. Each copy has the capability to correct the errors in other copy as the errors are almost never coextensive. In this approach the cost incurred is ~100,000 less compared with the first generation technologies in encoding and decoding information [20].

Challenges associated with this scheme include the cost which is unfeasible and the time for reading and writing onto DNA. However the cost associated with synthesizing and sequencing of DNA has been dropping at 5–12 exponential rates per year which is relatively much speedy than electronic media [20]. These can be leveraged in the future as DNA sequencers which are hand-held are recently available which fastens the reading process.

Church et al. have not paid their attention towards compression, parity checks, redundant encodings, and error rate. Therefore attention has to be focused towards error correction for the purpose of improving density and safety [20].

Hence to overcome this issue Goldman included improved base 3 Huffman coding instead of the one-bit-per-base representation [21]. This approach encoded a total of files of 730 KB including five types of computer files to emphasize the ability to store arbitrary digital information. They included 154 of Shakespeare's sonnets (ASCII text), a classic scientific paper (PDF format), a medium-resolution colour photograph (JPEG 2000 format), a 26 s excerpt from Martin Luther King's 1963 “I Have A Dream” speech (MP3 format) and a Huffman code to convert bytes to base 3 digits (ASCII text). Binary code was transformed to ternary code and then was represented in triplet DNA code. Fourfold redundancies were used to avoid data loss. To ensure security each redundant chunk was complemented in alternate chunk. This approach ensured high scalability and reliability. This paved the way towards high capacity and low maintenance data storage mechanisms as Goldman et al. were able to achieve a storage capacity of 2.2 PB/gram [20, 21].

*Encoding Scheme for Small Text Files.* Majority of the encoding schemes are performing superior for large text files. This approach is for small text files. This approach primes to high volume data storage density via dipping number of nucleotides for representation while depending on sequence transmission algorithms [22].

At present many loss less compression algorithms are in use but still they require ample context information for encoding purposes.

Burrow-Wheeler transformation (BWT) [23] has a decent compression ratio with adequate use of context information. But here in order to achieve this through small text, BWT was trailed by Move-to-Front transformation (MTF) [23].

The following three steps are performed to generate context information through this encoding scheme.

- Text file compression: Huffman coding method was employed for this. Output is a binary sequence set.
- Mapping function: Two nucleotide base pairs were effectively employed to represent four binary bits. Foundation for choosing 4 bits for 3 nucleotides is output of Huffman coding being a hexadecimal value.
- Encryption: For the purpose of maintaining security, encoded message ought to be encrypted. One Time Pad (OTP) which require a random key of same length as message encoded is employed for this.

This approach reviewed that maximum efficiency of compression is possible to be achieved through performing transformation prior to compression thereby reducing number of nucleotides. This directly affects reducing cost factor.

This scheme is more significant in military applications and signatures of living modified organism as they are small messages which required to be deposited for extensive period of time [22].

This research has not implemented the biological protocols to insert the sequence in genome of bacteria.

Future work to be addressed includes modification of transformation algorithm and designing other mapping function for encoding nucleotide sequence.

#### 2.1.2. Codes for Encrypting Data in DNA

Basically 3 codes have been used over the past to store information on DNA. All these codes generally considered that an alphabetic language is being encoded in DNA. Although most of the researches considered English as the alphabetic language, it could have been used for even shorthand, which is the writing scheme for phonetics.

For a code to be optimum it should satisfy the dual criteria as follows:

- It should use DNA (nucleotides) economically, mainly because synthesizing of extended oligonucleotides is an expensive process though replicating appears to be comparatively economical.
- It should be able to reconstruct the message after encoding of data.

Although it is not considered to be essential, if the coding scheme offers some error detection and protection mechanism it would be of tremendous advantage. But this feature is not considered vitally important, because there are other mechanisms for addressing this issue such as using multiple copies of DNA. As the written language inherently consists of self-correcting mechanisms it makes this feature of error detection and correction not essentially important [24].

*Huffman Coding.* This code uses the principle of varying the length of symbols used for representing a character. Most recurrently appearing character in the text is assigned the lowest number of symbols while the least recurrently appearing character is assigned the most number of symbols. Employing this principle leads to developing of a very economical code [25]. Average code length is around 2.2 characters in Huffman coding scheme. This is the least average codon length achieved [24].

Unambiguity of the code is achieved through comprising of only one way in which the encrypted message can be read once the starting point is mentioned [24].

Drawbacks associated with Huffman coding include not outfitting for numbers and symbols. This is mainly because the frequency of showing these symbols is highly dependent on the text which reviews the fact that they are unable to be included in formulating the Huffman code. Secondly it is not suitable for long term storage due to the fact that when different length codons assembled together it might not reveal a pattern. Therefore the future generations might not be able to detect the significance of the pattern [24].

*The Comma Code.* In this approach a single base (G) is considered as the comma. Codons of 5-base length are separated from each other using base G. % base codons consist of other three bases, namely, A, C, and T, further more limited to single A:T base pair and two G:C base pairs. The C of the second G:C pair is always positioned in the upper strand [24].

Consisting of isothermal melting temperature is the advantage of the composition of the message DNA utilizing this scheme. Dominant feature of comma code is the reading frame of six codons including G, the comma, which is not achieved by other codes. This helps to identify a clear reading frame without the necessity to mention a starting point. Protection mechanism from insertion and deletion mutation is also guaranteed by this approach which makes the other codes much more complex [24].

Drawback of this code is that it is not economical as it repeats the comma-base G to create an automatic reading frame [10].

*The Alternating Code.* This scheme consists of 6 base codons which are 64 in number including pyrimidines and purines. Construction of the message DNA in an entirely synthetic nature is the primary feature of this approach. As this creates fully artificial DNA it is suitable for long term storage which overcomes the drawback of Huffman code. In addition, it offers benefits such as being isothermal and error detecting but it is not superior to comma code [24, 26].

Alternating code also comprises repetitive features which makes it noneconomical [10]. It is the main drawback associated with this coding scheme. Therefore attention of the researchers has been led towards developing an economical code without repetitive features [10].

*Comma-Free Code.* It is also known as prefix free code. This comprises fixed length base frames without commas to separate the frames. Therefore, it uses an automatic frame detection mechanism. Comma-free code [24] does not consist of identical four base pairs which is the only way of hindering from natural DNA sequences. These codons are possible to be read simply in one way and support error detection mechanisms as well.

Although comma-free code is robust and the error correction works to correct against small-scale loss such as DNA point mutations, it does not have the ability to recover broken data when a large DNA segment is deleted from the data encoded DNA region [10].

*Improved Huffman Coding Scheme.* Each approach used for information storage in DNA differs in the economical use of nucleotides. Here Ailenberg and Rotstein use the principles of Huffman coding to define DNA codes for the entire keyboard, for clear-cut information coding [14]. This overcomes the drawback of the Huffman code, being limited only to the letters of the alphabet. This is based on a construction of a plasmid library with specially designed primers embedded along with the message for fast retrieval. Index plasmid only contains details about the structure of the information library. A good encoding scheme should have economical use of nucleotide per character which is about 3.5 here (base-to-character ratio) [14].

Other coding schemes have low base-to-character ration but is limited to lower number of characters such as the English alphabet. DNA was inserted into living organisms and they are subject to losing information due to breakage by mutation, insertion, and deletion. Hence, this approach is a solution to this problem as it is able to recover data of damaged DNA. Therefore this method overcomes the drawback of the comma-free code. This is also able to identify any frame shift due to mutation or errors in sequencing. This method uses unique primer design using plasmid DNA libraries [14].

*The Perfect Genetic Code.* In this approach Doig points out the fact that through varying the codon length efficiency of the code can be increased significantly [27]. Here Doig uses a variable codon length through employing more frequent amino acids with shorter codon length while rare acids are represented using a longer codon length. The only approach which is more efficient than this is using overlapping genes [27].

According to Doig the necessity for a fixed codon length comprising 3 bases requires 42% more DNA than the minimum requirement [27]. Perfect genetic code is 70% more efficient than the other codes due to the use of a variable code length. Doig employs the Shannon-Fano coding scheme to derive the binary code [27].

As there is very little redundancy, any mutation would cause a change in amino acids. Additionally, if the mutation is chief to fluctuation of codon length many of the mutations will be extensive frameshift mutations. According to Doig, it is impossible for the shift towards use of perfect genetic code, considering a simple example that Val has been coded for four codons comprising 3 bases. As the third does not convey any information it would be effective to code Val employing two bases only. The difficulty of machinery to shift from fixed codon length to variable codon length plus the priorly mentioned drawbacks leads to not using this effective code though it maximizes the efficiency through using variable length codons [27].

#### 2.1.3. Basic Approaches for Designing DNA Codons

There is no standard structure in which code words are generated due to the fact that importance of constraints to be addressed differs according to the encoding models [11].

*Template Map Strategy.* Codon's group proposed dividing the constraints on code words into two binary codes, namely, template,specifying GC content and maps indicating mismatch between word pairs. The product of the aforementioned codes results in a quaternary code. Later on Arita using the Hadamard code expanded this for longer code words with the mismatch beyond half the length of the code [11]. Downside of this is that melting temperature of the words may differ without considering the unvarying GC content. Comma freeness is another problem associated with this approach. Enlarging into multiple words will be an issue due to mishybridization [11].

*De Bruijn Construction.* Arita overcomes the problem of melting at different temperatures and also showed an ideal algorithm for choosing oligonucleotides to get rid of the higher risk of mishybridization because of the matched base pairs which are placed consecutively [11]. Disadvantages associated with this approach are small number of mismatches between words and comma freeness.

*Stochastic Method.* This is a widely used method in the recent past. Kac employed generic algorithms to find similar melting temperature code words. Complexity of the problem led them to apply genetic algorithms only to code words up to length 25 [7]. Arita could increase the code words in number designed by the template map strategy. This approach fails if it is started from the scratch which reveals the fact that it is better to use this approach to enlarge the code words of already designed word sets [11].

#### 2.1.4. Data Storage Style

Data storage style is the manner in which DNA words are stored in a medium. In these two approaches which are discussed in detail, DNA words are stored in solid and liquid media. Data storage style depends on the word design. DNA chips which are an immobilization technique had been popular among the public as the weaker constraint on words limits the design problems. A systematic word design which avoids mishybridization serves both surface based approach and soluble approach [11]. 

*Surface Based Approach.* DNA words are placed on a solid approach which is also known as solid phase approach. Advantages associated with this approach are that code words (codons) can be separated from the complements as single strand of the double helix structure is powerless, which in turn reduces the risk of unforeseen combination of words and easiness to recognize the words separately for the purpose of information reading due to effective fluorescent labeling [11].

*Soluble Approach.* Benefits associated are opening up of the possibility for independent information processing and possibility for introducing DNA words into microbes. Disadvantages include limiting inborn abilities of molecules through providing easier access to information [11].

### 2.2. DNA Secret Writing

Secret writing is used to prevent illegal access of information by unauthorized parties. Cryptography and steganography are two methods used for secret writing. Cryptography manipulates information for misunderstanding while steganography hides the existence of information [4]. Traditional cryptography and stenography techniques have been identified as losing power and are becoming breakable as they are based on difficult mathematical problems which are mature both in theory and realization. Therefore researchers are investigating developing hybrid cryptosystems involving DNA methodologies into cryptography and stenography. Hiding data in terms of DNA sequence is referred to as DNA cryptography [28]. Data embedding is also referred to as stenography or DNA watermarking. Adleman's research [29] and research on hiding messages in microdots [30] are the birth of DNA stenography and cryptography. DNA data embedding is made possible via

- DNA methods: writing into DNA using insertion and creation;
- DNA sequencing: reading DNA.

Objectives of cryptography are mainly as follows.

- Authentication: Authentication is confirmation of the details about entity from which we are receiving information. Digital signatures, passwords, and trademarks are used to ensure authentication.
- Data confidentiality: This is the process of securing confidential data from unauthorized personnel. In cryptography this goal is achieved through encryption.
- Data integrity: This is to guarantee that information is received in the exact format in which it has been sent by the official party. That includes the fact that no modification or alteration is done during cryptography process [31].

Practical methods for DNA data embedding are twofold:

- DNA based stenographic methods are proposed by Bancroft and Clelland by physically secreting information in a living organism so that PCR and secret key are essential for retrieving information [32].
- Cox proposed embedding information in living beings so that information will be carried by organism along with cell replication without affecting biological properties of living organism. This can be done in two ways [33, 34].
  - Replacement of DNA in noncoding segments never being transferred to proteins: drawback is that extreme care is needed to ensure that this insertion would not affect the biological functions.
  - Modifying coding DNA (cDNA) partitions which get transferred to proteins: this approach is more systematic and safer [11].

#### 2.2.1. Problems Associated with DNA Cryptography

Nonavailability of a hypothetical basis and lack of knowledge associated with DNA cryptographic methods are major problems. Similarly high cost and difficulty in understanding also have effect, in addition to inappropriateness to be used by general public due to the biological tests and trials which have to be performed in highly technology equipped laboratories.

#### 2.2.2. DNA Secret Writing Algorithms

*Steganography Technique Using DNA Hybridization.* This method effectively uses the advantages offered by the structure of DNA for vast storing capabilities and parallel molecular computation. This approach brings out an algorithm for hiding data in DNA in a digital form [35, 36].

**Figure 2:**
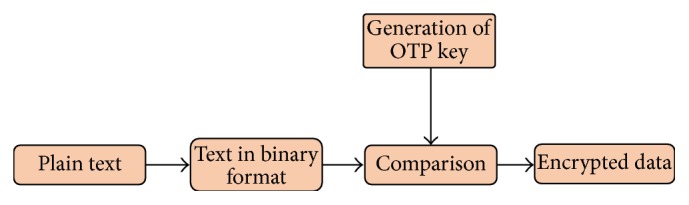
Block diagram of encrypting a message using DNA hybridization technique.

One Time Pad (OTP) generated keys are used as encryption key. This key is used just only once for exactly one message. The used pad is destroyed by the user after encryption.  Figure 2 summarizes the encryption process using hybridization technique.

**Figure 3:**
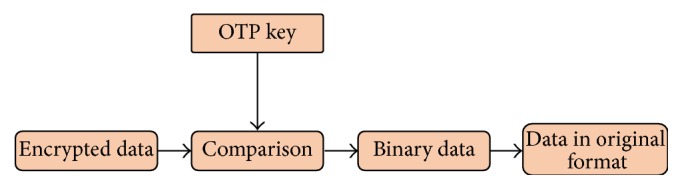
Block diagram of decrypting a message using DNA hybridization.

After decrypting the message receiver destroys the identical pad which is owned by him. Because of this reason this approach is extremely secure. In this algorithm single stranded DNA is used as the OTP. Length of the OTP should be 10 times larger than the binary message [35]. Encrypted message is hidden in a microdot. Strand consisting of the encrypted message is placed between two PCR primer sequences. Then it is hidden in many similar structures. Without the knowledge about start and end primers one will not be able to read the message by amplifying. In reading the message: hybridized segments are read as “1” and unchanged ssDNA segments are read as “0” [3].  Figure 3 is a diagrammatic representation of the decryption process using DNA hybridization technique.

DNA hybridization is a slower process at the beginning because it is difficult for two complementary strands to combine together. But later this is a rapid process. This can be effectively utilized in searching and parallel computation. Restrictions at present for this process are time consumption and expansiveness [35].

So, it already provides an honest security and takes solely less time for the message to be communicated [37]. 

*Chromosomes DNA Indexing.* In this approach* Homo sapiens'* chromosomal sequence is used as the OTP key for the process of encryption and decryption. Plain text is converted to ASCII code and then to binary code and lastly concerted into DNA format. OTP key of* Homo sapiens* is taken and scanned to examine the DNA form of plain text in the OTP key. For each and every character in plain text found in the chromosome an array of indexes is formed placing the starting point of the index in chromosome using DNA indexing method [4].

**Figure 4:**
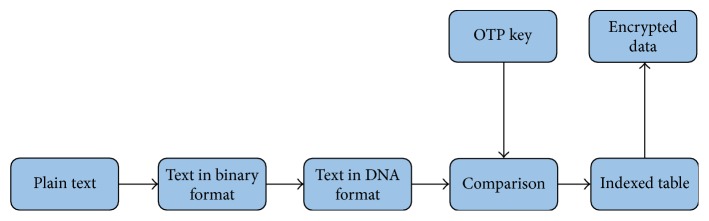
Block diagram of encryption process using DNA Chromosome Indexing.

**Figure 5:**
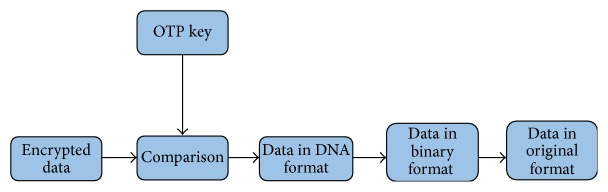
Block diagram of decryption process using DNA Chromosome Indexing.

Diagrammatic representation of the encryption process by chromosome DNA indexing is represented in Figure 4 while Figure 5 details the decryption process by chromosome DNA indexing. Communication of primers, chromosomes, and OTP is a major difficulty in cryptography. This algorithm overcomes this problem as only the types of chromosome and primers need to be known in advance.

This algorithm uses the vast randomness of the DNA medium. This is the downsize of this approach. This cannot be termed as a proper cryptographic algorithm due to this fact. Hence, OTP key can be used only once [35].

*DNA XOR OTP with Tiles.* This is based on DNA tiles and DNA XOR tiles of Biomolecular Computation (BMC). Tiles carrying the message are binding together in a string. Then an XOR operation is performed between the plaintext and the OTP which is the encryption key. Tiles possess two pairs of sticky ends one to bind between tiles containing the message and the other to bind to OTP tiles. Restrictive enzymes are used to remove the cipher text from the rest of the tiles.

In decryption process OTP is used to extract the encryption key by the user. Self-assembling of the tiles used for encryption in reverse order results in giving the plain text [36–38].

### 2.3. DNA as Organic Data Memory

Incorporating DNA into a living host, who has the ability to withstand risky ecological conditions, has the ability to grow rapidly, and is able to tolerate addition of artificial gene sequences was, the solution proposed for generating a reliable storage medium. Inoculating DNA sequences into an organism is a challenge because it is difficult to retrieve a message from a whole organism composed of many genomes. Another obstacle is the unpredictable nature of genomic mutation [39].

DNA memory prototype consists of 4 main steps:

- Encoding information as artificial DNA sequences.
- Injecting the sequences to living organisms.
- Permitting the organisms to be nurtured.
- Extracting information back from organisms.

#### 2.3.1. Memory Application of DNA

DNA memory can be effectively utilized in commercial applications and in national security for information hiding purposes and for data stenography.* Deinococcus* bacteria can live and multiply without human interference [39]. This property can be used to preserve data at nuclear catastrophe [14].

There exists a competition among seed companies to protect their investments. Therefore, incorporating a DNA watermark in the seeds could be a better approach to track their sales and preserve their copyrighted products against illegitimate planting [39]. This would not affect the farmers who are in need but covetous farmers.

In order to capture pollutants that will contaminate with the ecological resources and will pollute the resources, researchers drill wells to gather samples of soil. In cooperating sufficient information in bacteria which could update the current status of soil with time uninterruptedly by tracking bacteria's distribution spatially and temporally, using developed technologies would be an effective approach for this purpose [39].

Endangered species could be identified by injecting a DNA watermark into the genome of the subject, replacing the other synthetic identification. This could also be used to preserve safely the personal information of a person such as medical information and family history in their own bodies [39].

### 2.4. Big Data Storage in DNA and DNA Computing

Era of DNA computing begins with the identification of limitations in electronic computers. The volume of data that can be stored in an electronic computer and the speed thresholds that can be reached which is governed by the physical characteristics of computers are the main limitations identified in big data storage [40]. DNA computer addresses the above-mentioned limitations through solving computational problems engaging molecule manipulations while discovering natural computational models leading to the big data storage and big data analytics in DNA.

Advantages offered by DNA computing include consuming significantly less energy than the electronic computers. Energy consumed by DNA computers is billion times comparatively less than other electronic computers. The storage space needed to store information is less than trillion times over electronic computers [40]. Furthermore DNA computers offer parallelism at a high level. Millions and trillions of molecules perform chemical reactions parallel [40].

#### 2.4.1. Hamilton Path Problem

Adleman addresses the Directed Hamilton Path problem through exploring possibilities of information encoding in DNA sequences and thereafter performing simple operations for strand manipulations. The simplified version of this problem was the salesman problem, which needs to find out the optimal path out of a pool of cities through which salesman has to travel. Adleman complicated this problem through restricting the connection routes between cities and specified the start and end of the journey.

This approach solves the above-mentioned problem with the intention of generating random paths through the graph where Adleman encoded each node in the graph into a random strand comprising 20 bases. Each and every edge of the graph was represented by another different oligonucleotide consisting of 20 bases complementary to the source node's second half and target node's first half. This results in self-assembling and ligation of compatible edges by the function of T4 DNA ligase enzyme. In order to filter the paths the product of the self-assembling process is subjected to amplification by PCR. To filter the paths with the exact length, separation and recovery of DNA strands with the exact length are done by Agarose gel electrophoresis. Agarose gel electrophoresis [41] is the identified most effective way of separating DNA fragments constituted of variable lengths. Prior to the employment of Agarose gel for separation of DNA fragments, it was done using sucrose density gradient centrifugation. It only provided a separation based on the approximation of size [41]. Filtering out the paths which cover all the nodes is achieved through affinity purification successively for all nodes. In order to check the presence of the path, PCR was employed to check the existence of a molecule encoding a path of Hamiltonian [27, 40].

The time duration required for the practical approach was around 7 lab days. Adleman's algorithm was more labour extensive. Process automation could address the problem of high labour intensity. The algorithm used here was inefficient as the number of oligonucleotides needed increased linearly with the increase of edges and exponentially with the number of vertices. This is energy efficient. It is not clear whether the large number of inexpensive operations could be used for resolution of real computational problems [27]. Speed of a computer is measured by two factors.

- The number of operations it can perform parallel.
- How many steps each process can perform per unit time.

With regard to first measure it is in favour of DNA computers because of the vast parallelism it offers. Second measure is in favour of electronic computers because considering a personal computer, it can perform 100 million instructions per second [42]. But as the parallelism offered by DNA computers is massive, time for executing one instruction is not problematic.

With the enormous flexibility offered by electronic computers through the numerous operations it is pretty efficient to multiply two 100 digit numbers whereas it is overwhelming to perform this using a DNA computer with the protocols and enzymes presently available [27].

#### 2.4.2. 20-Variable 3-SAT Problem

This is the largest problem solved yet with a DNA computer which is 20 variables for three-satisfiability problem. This problem is an NP (Nondeterministic Polynomial) time-complete computational problem. As the problem complexity is very high, even with fastest sequential algorithms, exponential time to solve this problem is required [43]. This was the reason it leads to examining the performance of DNA computers. Subsequences based separation used in Sticker Model [44] is used to solve this problem. Two basic operations are used by Striker Model for computations: application of strikers and separation based on subsequence. 20-variable SAT problem uses separations. Oligonucleotide probes restrained in polyacrylamide gel-filled glass modules are employed for carrying out separations. Electrophoresis moves the DNA strands through the module. Strands which are complementary to the immobilized modules hybridized while the strands which do not contain complementary strands pass by. Electrophoresis at a higher temperature which is higher than the melting temperature of the probes is used to free netted strands. Remaining strands are transported to other modules [41]. This problem maximizes the use of parallelism offered by DNA computers.

## 3. Results and Discussion

DNA has been identified as the potential medium for data storage due to its vast storage capacity, high data density, sustaining to extreme environmental conditions, and so forth. Evolution of data storage in DNA is described in detail in this review article. Idea of data storage in DNA emerges with Microvenus project of Davis [5, 6]: to store an image to DNA leads the foundation for DNA based storage system with the core idea of storing abiotic information on DNA. Genesis project [5, 7] succeeded the Microvenus project. Microvenus was inaccurate as it was not uniquely decodable while Genesis was also inaccurate and was inefficient because it did not return the original text after decoding. Although DNA has been identified as the potential medium for long term data storage there these issues were identified as the hitches to be addressed: high cost of DNA synthesis, data read back at a slower rate, DNA not possessing rewriting capabilities, and DNA not allowing random access. Among the above identified issues Yatchi et al. have been able to overcome the issue of slower data read back speed using alignment based encoding model [10]. Rewritable and random access based storage systems [13] proposed by Tabatabaei et al. overcome the issue of not allowing random access and rewriting. Comparison of encoding models used is summarized in Table 1.

PCR based encoding models [5] are highly secure. Main drawback associated with PCR based models is scalability of data. Yachie et al. proposed iDNA [8] method to overcome scalability problem. Drawbacks of requisition of PCR, knowledge on primers, and insertion of errors in the template region which results in impossibility of recovering the data have not been addressed. Therefore researchers searched for a PCR independent encoding model.

Alignment based encoding model [10] achieves the advantage of not needing a template DNA. It performs retrievals without parity checks or error correcting algorithms. Though this approach efficiently retrieves data from a genome, it requires sequencing of the entire genome which is the main downside associated with this approach. High speed and lower cost of reading are identified in this approach. As the data could be lost during evolution of the living organisms, Yatchie et al. [10] introduced multiple copies of sequences compressed by multiple compression paths, though this factor overcomes the data loss during evolution and it leads to redundant volumes of data. Data recovery rate is fragile in this approach. Size limit of the oligonucleotides is a downside of this approach as if the size is increased it might appear in the original genome.

Church and Goldman model [12, 14] marked a milestone in DNA storage as all the prior work was only for small volumes of data. This algorithm efficiently uses bases as it encodes one bit per base. Significant drawback associated here is the lack of error correction mechanism. Next generation digital information storage techniques employ one-bit representation per base. Cost associated and time for reading and writing data are main drawbacks of this approach. Aforementioned drawbacks could be accessed in the near future as hand-held reading devises are becoming available. Church et al. have not paid their attention towards compression, parity checks, redundant encodings, and error rate. Attention has to paid towards improving the storage density and safety. Placing multiple copies of data would lead to increasing the safety measures.

Numerous encryption schemes have been used to encrypt data in DNA. Huffman code [24, 25] has been developed for a limited number of symbols only the English alphabet. This drawback has is overcome by the improved Huffman code [14] by defining the code for the entire keyboard, images, and music. Comma code uses base G to identify and separate the reading frames. Both comma code and alternating code use repetitive bases to separate reading frames. Comma-free code has an automatic frame detection mechanism through which it overcomes the drawback of redundancy. Alternate code is not superior to the comma code. Although perfect genetic code [27] varies the codon length which in turn increases the efficiency a paradiagram shift is needed to move into perfect genetic code as machines need to read variable length codons. Comparison of the encryption schemes used is summarized in Table 2.

DNA is effectively used for secret writing due to the security mechanisms offered by DNA. DNA cryptosystems are more secure due to the huge size of the OTP key used for encryption. It will be extremely hard to break the algorithm without knowing the primer sequences and scientific specifications of the organism. Running time of cryptographic systems is less. Larger key and the indexes of arrays used in DNA systems need high memory space. Therefore in practical situations it might require a separate storage device. In the hybridization method, an OTP key 10 times bigger is generated for each binary bit of plain text. In DNA indexing the key obtained from the public database is extremely more huge than the hybridization method. Encrypted data is obtained by scanning a randomly picked number from the key and the plain text of DNA form. Random pick of numbers along with the lengthy OTP key improves the confidentiality. Implementation of cryptographic systems is highly costly.

In order to provide more security, OTP key generated in the hybridization method can be increased in length further. It is possible by generating an OTP key of more than 10 bases in length (say 12 or more). It can be concluded that “the higher the length of the key data, the higher the security.”

In order to enhance the security of cryptographic systems further step of hiding the data after encryption could be practiced. It can be achieved through performing the biological process of hiding the encrypted data between the primers in the DNA sequence. Comparison of the performance of the basic cryptography algorithms is summarized in Table 3.

Incorporating messages into human, mouse, or bacterium was popular but the challenges imposed were extracting the information from the whole genome without knowing any tracks about the embedded message and the unpredictable nature of genomic mutations. Wong et al. [39] overcome this challenges through identifying fixed size sequences that usually do not exist in the living genome. This is the critical operation as they do not want to cause any damage to the living genome. Mutation of the organisms is a major challenge, influencing the integrity of the messages in this approach. Selecting organisms with low mutation rate would be a possible solution to overcome it.

Cost and the data retrieval rate are major problems in DNA based storage systems. At present data can be read at a rate about 100 MB per second by storage devices which is much higher rate than natural storage. Synthesis and sequencing processes are time consuming and require expertise knowledge which makes this method inaccessible to general public. Even though DNA is scalable, robust, and stable the above-mentioned drawbacks have a high concern. Making a custom DNA molecule is expensive and this has been identified as the major obstacle for DNA based storage systems [45]. Recently parallel automation and decreasing the cost of synthesis using next generation sequencing techniques are in operation. Therefore, simplified purification techniques are a major area to be focused. At present researchers have focused on developing DNA storage system which is consisting of reading and writing chambers [8] as the initial step towards developing a molecular data storage system. To ensure security the researchers have translocated the information to safety zones known as parking spots. In order to read DNA, it has been transferred to coding stations using electric field gradients, electronic motors, and so forth. In order to make molecular storage device a commercial application researchers have to focus on scaling natural storage capacity. High time consumption associated with the overall general process of encoding, amplifying, sequencing, restructuring, and decoding is also a major challenge. Many errors such as homopolymers, sequencing errors, lower access rate errors are available. Though autocorrection mechanism is available in natural DNA there is no such mechanism in synthetic DNA. Future work includes analyzing the environmental conditions under which the experiments are carried out as well as preservation of DNA for long term storage.

## 4. Conclusion

This review article critically analyzes the existing methods of storing data onto DNA. Data is encrypted into DNA using diverse codes and this article analyzes and discusses the codes used for encrypting data. Multiple approaches for designing DNA codons and diverse data storage styles have been analyzed in detail identifying the pros and cons of each approach. Secret writing techniques using DNA molecules for secure data storage are also discussed through this article. DNA can be used as an organic memory to store massive amounts of data. This paper also analyzes the mechanism where living organism could be used as storage devices while identifying limitations and appropriate applicability. Challenges faced through trying to apply organic memory concepts are also discussed through this paper. Big data storage and analytics and the way it has led to DNA computing to solve hard computational problems are also discussed here. The outcome of this study is a review article which identifies the limitations of existing encoding algorithms and proposes methods to overcome the identified limitations.

## Figures and Tables

**Table 1:** Comparison of encoding models.

Encoding model	Advantages	Disadvantages
Microvenus project	Laid the foundation for storing abiotic information in DNA	Being inaccurate and not distinctively decodable

Genesis project	Laid the research work to explore the intricate relationship between biology, belief systems, information technology, dialogical interaction, ethics, and the Internet	Inaccurate as the original sentence was altered during mutation at the presence of ultraviolet light

PCR based encoding models	High security because of the size of the microdots and even if an adversary identifies the microdot it would be extremely difficult without the knowledge of the primer sequence	Insertion of errors in template region making it unmanageable to recover the encoded data Need of the knowledge of primers Widespread experimental obstacles and practical problems Need of PCR Data breakage that could occur in encoding and decoding procedures due to errors of humans

Alignment based encoding models	Independent of Polymerase Chain Reaction Greater speed and lower cost of reading DNA data and lower cost of synthetic DNA Positions of the data breakages that could be identified easily by the alignment results although they were not recoverable	Multiplication of cassettes leads to redundant volumes Parity effects cost a certain volume of data sequence Data recovery rate is fragile and is proportional to data breakage which occurs through DNA deletion of long ranges Sequencing of the entire genome is required to retrieve data There is size limit of the cassette oligonucleotides being used to encode the message. If it increases a certain limit there is a possibility of it to appear by chance in host genome

Rewritable and random access based DNA storage system	Random access to data blocks of DNA which promotes nonlinear access Rewriting capability of information into random locations Cross hybridization problems that are eliminated in this method by prohibiting redundancy of information Being used to store frequently updated data which needs to memorize the editing history	High cost

Next generation digital information storage	Employment of one-bit representation per base High scalability High data storage density Highly reliable Each copy having the capability to correct the errors in the other copy as the errors are almost never coextensive	Cost is unfeasible Time for reading and writing onto DNA is high

Encoding scheme for small text files	High volume data storage density Not needing ample context information for encoding purposes Maximum efficiency of compression Reducing cost factor	Have not proceeded in implementing the biological protocols to insert the sequence in genome of bacteria

**Table 2:** Comparison of encryption codes.

	Huffman code	Comma code	Alternate code	Comma-free code	Improved Huffman code	Perfect genetic code
Base-to-character ratio	~2.2	~6	~6	Variable	~3.5	Variable

Economical	Very economical	No	No		Yes	

Long term storage	No	Yes	Yes			

Error correcting			Yes	Yes	Yes	

Protection from mutation		Yes			Yes	No

Isothermal melting temperature		Yes	Yes			

Synthetic DNA		Yes	Yes			

Special features	Uses the principle of varying the length of symbols used for representation based on the recurrence of a character Not applicable to numbers and symbols	Consists of fixed length reading frames of 6 bases including the comma, G		Fixed length base frames without commas to separate the frames Does not consist of identical four bases Does not have the capability to recover the broken data when a large segment is deleted from a data encoded region	Stores text, images, and music in DNA Expensive	70% more efficient than the other codes due to the use of a variable code length

**Table 3:** Comparison of the performance of cDNA secret writing techniques.

Features	DNA hybridization technique	Chromosome DNA indexing
Running time	Less	More

Size of the key	Large depending on the input	Large independent of the input

Strength of the algorithm	High based on the type, size, and the randomness of the key	High based on the key type and key size and the randomly produced index

Memory space	Needs more memory space for storing the lengthy key and performing the operations involving it	More than the hybridization type because of the huge key length and the index array involved

Cost	High	High

Longevity	Believed to withstand any duration	Believed to withstand any duration
